# Pharmacokinetic behaviour of the chemoprotectants BNP7787 and mesna after an i.v. bolus injection in rats

**DOI:** 10.1038/sj.bjc.6601719

**Published:** 2004-03-16

**Authors:** M Verschraagen, E Boven, E Torun, C A M Erkelens, F H Hausheer, W J F van der Vijgh

**Affiliations:** 1 Department of Medical Oncology, Vrije Universiteit Medical Center, De Boelelaan 1117, 1007 MB Amsterdam, The Netherlands; 2 BioNumerik Pharmaceuticals, Inc., 8122 Datapoint Drive, San Antonio, TX 78229, USA

**Keywords:** BNP7787, mesna, pharmacokinetics, plasma, kidney, tumour

## Abstract

In preclinical studies, BNP7787 (disodium 2,2′-dithio-bis-ethane sulphonate), the disulphide form of mesna, has demonstrated selective protection against cisplatin-induced nephrotoxicity due to conversion into mesna inactivating toxic platinum species. Mesna (sodium 2-mercapto ethane sulphonate), however, can affect the antitumour activity of cisplatin, while BNP7787 does not interfere with the antitumour activity. To understand the difference in interference with cisplatin-induced antitumour activity between BNP7787 and mesna as well to characterise the selective nephroprotection by BNP7787, the pharmacokinetics of BNP7787 and mesna, each given i.v. 1000 mg kg^−1^, were determined in plasma, kidney, liver, red blood cells (RBC), skeletal muscle and tumour of Fischer rats bearing subcutaneously implanted WARD colon tumours. The following results were obtained: (1) high concentrations of BNP7787 and mesna were observed in the plasma and kidney after administration of BNP7787 or mesna, except for mesna in plasma after BNP7787 administration; (2) in all other sampled compartments, the AUC values of both compounds were at least 5.5-fold lower than the corresponding values in kidney; (3) the AUC of mesna in plasma after mesna administration was comparable to the AUC of mesna in kidney after a dose of BNP7787 that can completely prevent cisplatin-induced nephrotoxicity in rats; (4) the AUC of mesna in plasma was five-fold higher relative to the AUC of mesna following BNP7787 administration (*P*<0.01). In conclusion, the five-fold higher AUC of mesna in plasma after mesna administration and the fact that mesna is more reactive with (hydrated) cisplatin than its disulphide form BNP7787 represent a plausible explanation as to why mesna administration can reduce the antitumour activity of cisplatin. After BNP7787 administration, the distribution of BNP7787 and mesna was restricted to the kidney, which confirmed the selective protection of the kidney by BNP7787.

Cisplatin (*cis*-diamminedichloroplatinum [II], CDDP) is a highly active cytotoxic agent that is widely used for the treatment of solid tumours, including those of the lung, head and neck, ovary and testis. Besides its antitumour activity, cisplatin can cause serious side effects, such as dose-limiting nephrotoxicity and neurotoxicity. Several chemoprotectors have been studied with a view to prevent or reduce cisplatin-induced nephrotoxicity. An optimal chemoprotector should meet the following requirements: it should protect against the nephrotoxicity and preferably also against other common and serious cisplatin-induced side effects; it should not reduce the antitumour activity of cisplatin and should not be toxic by itself (Hausheer *et al*, 1998). Various protective compounds, such as thiosulphate (Wagner *et al*, 1988), diethyldithiocarbamate (Berry *et al*, 1990) and amifostine (Hospers *et al*, 1999) have failed to meet these requirements due to side effects or interference with the antitumour activity of cisplatin.

Mesna (sodium 2-mercaptoethane sulphonate), which is used clinically to prevent oxazaphosphorine-induced haemorrhagic cystitis (Links and Lewis, 1999), has been investigated as a potential chemoprotector against cisplatin-induced nephrotoxicity (Kempf *et al*, 1985). Studies in tumour-bearing rats and nude mice, however, have demonstrated that mesna administration preceding cisplatin can reduce the number of complete and partial tumour remissions (Hausheer *et al*, 1998) and reduce the specific growth delay (Boven *et al*, 2002) in comparison with data obtained with cisplatin alone. In contrast, administration of the disulphide of mesna, BNP7787 (disodium 2,2′-dithio-bis-ethane sulphonate), in the same animal tumour models (Hausheer *et al*, 1998; Boven *et al*, 2002), did not reduce the antitumour activity of cisplatin. A likely explanation for the difference in interference with cisplatin-induced antitumour activity is a difference in the reactivity of BNP7787 and mesna with cisplatin in combination with a difference in concentrations between BNP7787 and mesna in plasma and tumour levels after administration of BNP7787 or mesna. After mesna administration, the plasma levels of the thiol compound mesna, which is approximately two-fold more reactive with (hydrated) cisplatin than BNP7787 (Leeuwenkamp *et al*, 1991; Verschraagen *et al*, 2003a), might be higher than after BNP7787 administration, which will result in inactivation of cisplatin in plasma before it can reach the tumour tissue. In addition, mesna concentrations after mesna in tumour tissue might be higher than after BNP7787 administration, resulting in local inactivation of cisplatin.

It has been previously demonstrated that BNP7787, which was nontoxic after i.v. administration in high doses (i.e. 2000 mg kg^−1^ in dogs and 4000 mg kg^−1^ in rats), completely protected against cisplatin-induced nephrotoxicity in rats and dogs based on plasma creatinine and BUN values, and more definitively by renal histopathological examinations, which were indistinguishable from normal healthy controls (Hausheer *et al*, 1998). The selectivity of BNP7787 is probably based on the preferential uptake of BNP7787 in the kidneys and the subsequent local formation of its metabolite mesna. *In vitro* studies using isolated cells have shown that the hydrophilic compound BNP7787 was preferentially taken up in intestinal and renal epithelial cells via facilitated transport (Ormstad and Uehara, 1982; Ormstad *et al*, 1983; Brock *et al*, 1984; Ormstad and Ohno, 1984). In these cells, BNP7787 was reduced to mesna (Ormstad and Uehara, 1982; Ormstad, 1983). The high concentrations of mesna in the kidney can locally inactivate (hydrated) cisplatin by forming nontoxic platinum complexes and can additionally protect against cisplatin-induced toxicity by acting as an antioxidant (Bast *et al*, 1987; Mashiach *et al*, 2001) (Figure 1

**Figure 1:**
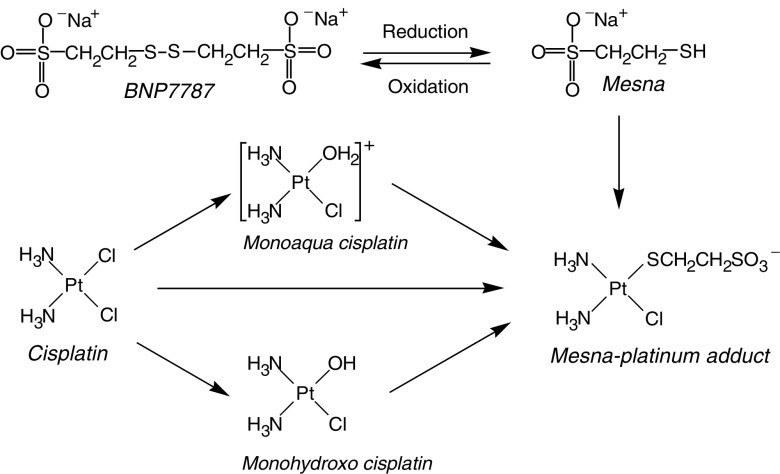
Proposed detoxification mechanism of BNP7787 in the kidney. After administration, BNP7787 will be converted into its metabolite mesna (and *vice versa*) in kidney, which can then react with cisplatin species to form inactive mesna–platinum adducts.

).

The first aim of this study was to find a possible explanation for the difference in interference with the cisplatin antitumour activity between mesna and BNP7787 administration. Therefore, the concentrations of BNP7787 and mesna were determined in the plasma and tumour tissue of WARD tumour-bearing Fischer rats after intravenous bolus injection of BNP7787 or mesna. Our second aim was to verify the selective protecting properties of BNP7787. Therefore, the concentrations and distribution of BNP7787 and mesna were measured in plasma, tumour as well as in kidney, red blood cells (RBC), liver and skeletal muscle. The liver was included, because it is an important excretion organ besides the kidney. Furthermore, the RBCs were included to determine whether they could act as a drug reservoir, whereas the skeletal muscle was included to investigate whether BNP7787 could be taken up in ‘normal’ (nonexcreting) tissue.

## MATERIALS AND METHODS

### Drug solutions

Drug solutions were prepared immediately prior to administration. BNP7787 was supplied by BioNumerik Pharmaceuticals (San Antonio, TX, USA) and was dissolved in water for injection at a concentration of 200 mg ml^−1^. Mesna was obtained from Sigma (St Louis, MO, USA) and was also dissolved in water for injection to obtain a concentration of 200 mg ml^−1^.

### Animals

The protocol was approved by the ethics committee for animal experiments of the Vrije Universiteit medical center in Amsterdam, The Netherlands. This committee strictly adheres to the standards required by the UKCCCR for the welfare of animals in neoplasia (Workman *et al*, 1998). Female Fischer rats (144–171 g) were obtained from Harlan (Indianapolis, USA) and were implanted subcutaneously in both flanks with fragments (2–3 mm diameter) of the WARD colorectal tumour (Cao *et al*, 1994). The pharmacokinetic study started at the time the tumours had reached a mean size of approximately 600 mm^3^ (range 250–900 mm^3^).

### Sampling

Tumour-bearing rats were treated with 1000 mg kg^−1^ BNP7787 i.v. or 1000 mg kg^−1^ mesna i.v. injected in the tail vein. Approximately 5 min before sample collection, the rats were anaesthetised by i.p. injection with 0.1 ml of a mixture of 75 mg ml^−1^ ketamine plus 5 mg ml^−1^ xylazine per 100 g weight. Blood was obtained by heart puncture at 0, 5, 15, 30, 45, 60, 90 and 150 min after i.v. administration of BNP7787 or mesna. Thereafter, the kidney, liver and tumour were removed by surgical dissection. In addition, at time points 0 and 30 min, skeletal muscle attached to the thighbone was also collected. At each time point three rats were killed. Due to the relatively large variation in mesna and BNP7787 plasma concentration at *t*=15 min after BNP7787 and mesna administration and *t*=45 min after mesna administration, an extra rat was included at these time points.

### Sample pretreatment

Immediately after collection, the blood samples, which were collected in cooled EDTA-containing tubes (potassium-EDTA 1.6 mg EDTA ml^−1^ blood; Sarstedt, Germany), were centrifuged at 4°C for 10 min at 4000 **g**. Plasma was separated and immediately deproteinised by adding one volume of sulphuric acid (0.33 M) and one volume of sodium hexametaphosphate (5 g/100 ml). After vortexing and centrifugation for 2 min at 12 000 **g**, the supernatant was stored in aliquots at −80°C until analysis. One volume of RBCs was washed with 1 volume of cold phosphate-buffered saline (PBS) of pH 7.4 containing sodium phosphate (10 mM) and NaCl (140 mM; Hospital Pharmacy, VUmc, Amsterdam, The Netherlands). This mixture was centrifuged for 2 min, 12 000 **g** at 4°C. After removing the PBS, the remaining RBCs were stored in liquid nitrogen until further processing.

The kidney, liver, tumour and skeletal muscle were washed with cold PBS before storage in liquid nitrogen. After collection of all the samples at the different time points, tissue homogenates of kidney, liver, tumour and skeletal muscle were prepared by pulverisation using a Braun Micro-dismembrator (Salm and Kipp; Breukelen, The Netherlands) according to the method described by Peters *et al* (1986). An appropriate volume of aqueous EDTA (1 g l^−1^) was added to the RBC and tissue homogenates to obtain a concentration of 100 mg tissue (wet weight) per ml. After sonification (10 min) and vortexing (10 min), the samples were subsequently deproteinised by adding 1 volume of cold sulphuric acid (0.33 M) and 1 volume of cold sodium hexametaphosphate (5 g/100 ml). After thorough vortexing and centrifugation (2 min at 8500 **g** at 4°C), the supernatants were stored in aliquots at −80° until analysis.

### Sample analysis

BNP7787 and mesna concentrations were determined selectively in one run using micro-HPLC with a dual electrochemical detector (dual ECD) (Verschraagen *et al*, 2003b). Briefly, citrate-NaOH buffer (40 *μ*l of a mixture of 1 ml 5.0 M sodium hydroxide and 4 ml 0.5 M tri-sodium citrate buffer, pH 3.0) was added to 200 *μ*l of deproteinised plasma, RBC or tissue sample, and the sample was injected into the micro-HPLC system which consisted of a Phenomenex C18 guard column (10 *μ*m, 4 × 2.0 mm) and a Phenomenex Prodigy C18 ODS-3 column (5 *μ*m, 100 × 1.0 mm; Bester, Amstelveen, The Netherlands). The mobile phase consisted of tri-sodium citrate dihydrate (0.15 M), tetrabutyl ammonium dihydrogen phosphate (TBAP) (0.05 or 0.1 mM) and cysteamine (0.1 *μ*M), and was adjusted to pH 3.5 with 85% *o*-phosphoric acid. The flow rate was 0.05 ml min^−1^ and column temperature was 36°C. After separation of BNP7787 and mesna by micro-HPLC, both compounds were detected with a dual ECD (Decade with Twin option set at 36°C; Antec Leyden, Zoeterwoude, The Netherlands). At the first electrode, the disulphide BNP7787 was reduced to mesna by a reactor cell with a glassy carbon-working electrode (−1.6 V *vs* Hy-REF). At the second electrode, which consisted of a gold wall-jet electrode, the mesna generated from BNP7787 and the mesna already present in the samples were detected (+0.85 V *vs* Ag/AgCl).

The assays for BNP7787 and mesna in plasma were linear over the range of 15–1200 and 3–120 *μ*M, respectively. Quality control verification samples of BNP7787 and mesna in plasma were used at three different concentrations, that is, for BNP7787 45, 225 and 900 *μ*M and for mesna 4.5, 22.5 and 90 *μ*M. The RBC and tissue samples from the rats were measured with a calibration curve produced in tissue homogenate from pig kidney. In tissue, the dynamic range was 20–2000 nmol g for BNP7787 and mesna. Quality control samples of both compounds in tissue were used at three different concentrations, that is, 50, 250 and 1250 nmol g^−1^ for BNP7787 as well as for mesna. The lower limit of quantification of both compounds was 3 *μ*M in plasma and 20 nmol g^−1^ in tissue. The within- and between-day accuracy and precision for plasma and tissue were within 14 and 7%, respectively.

### Pharmacokinetic and statistical analysis

Semilogarithmic concentration–time curves of BNP7787 and mesna were obtained by plotting the mean concentration (*C̄*_*i*_) of each drug *vs* time (*t*_*i*_). Areas under the concentration–time curves (AUCs) were calculated over 5–150 min after administration of the drugs or up to the time of the last measurable concentration (>LLQ) before 150 min after administration. AUCs were calculated by the trapezoidal rule:


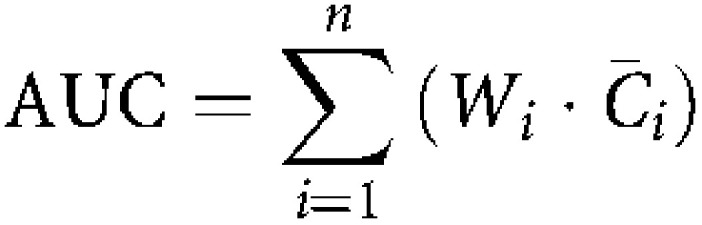


with


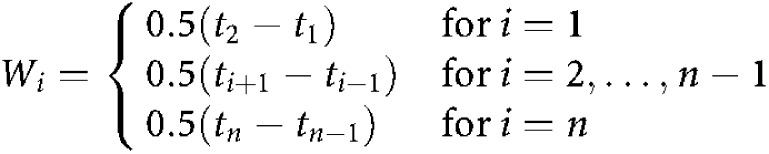


The standard errors (S.E.) of the AUC values were calculated by:


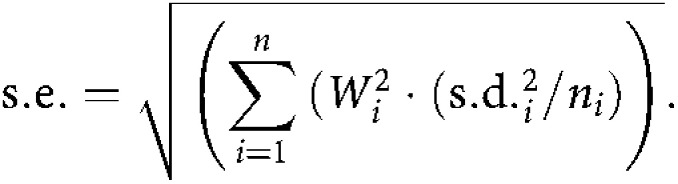


in which s.d._*i*_ is the standard deviation of the concentrations of the drug at each time point *i* and *n* is the number of animals at each time point *i*.

The *z*-test was used for the statistical evaluation of the results, as suggested by Bailer for destructive measurement techniques (Bailer, 1988). Since several hypotheses were tested, the *P*-values were adjusted for multiple pairwise testing using the Bonferroni formula: adjusted *P*=k^*^P, in which *k* represents the number of comparison tests (Altman, 1991). *P*-values less than 0.05 were considered statistically significant.

## RESULTS

### Mesna administration

**Figure 2:**
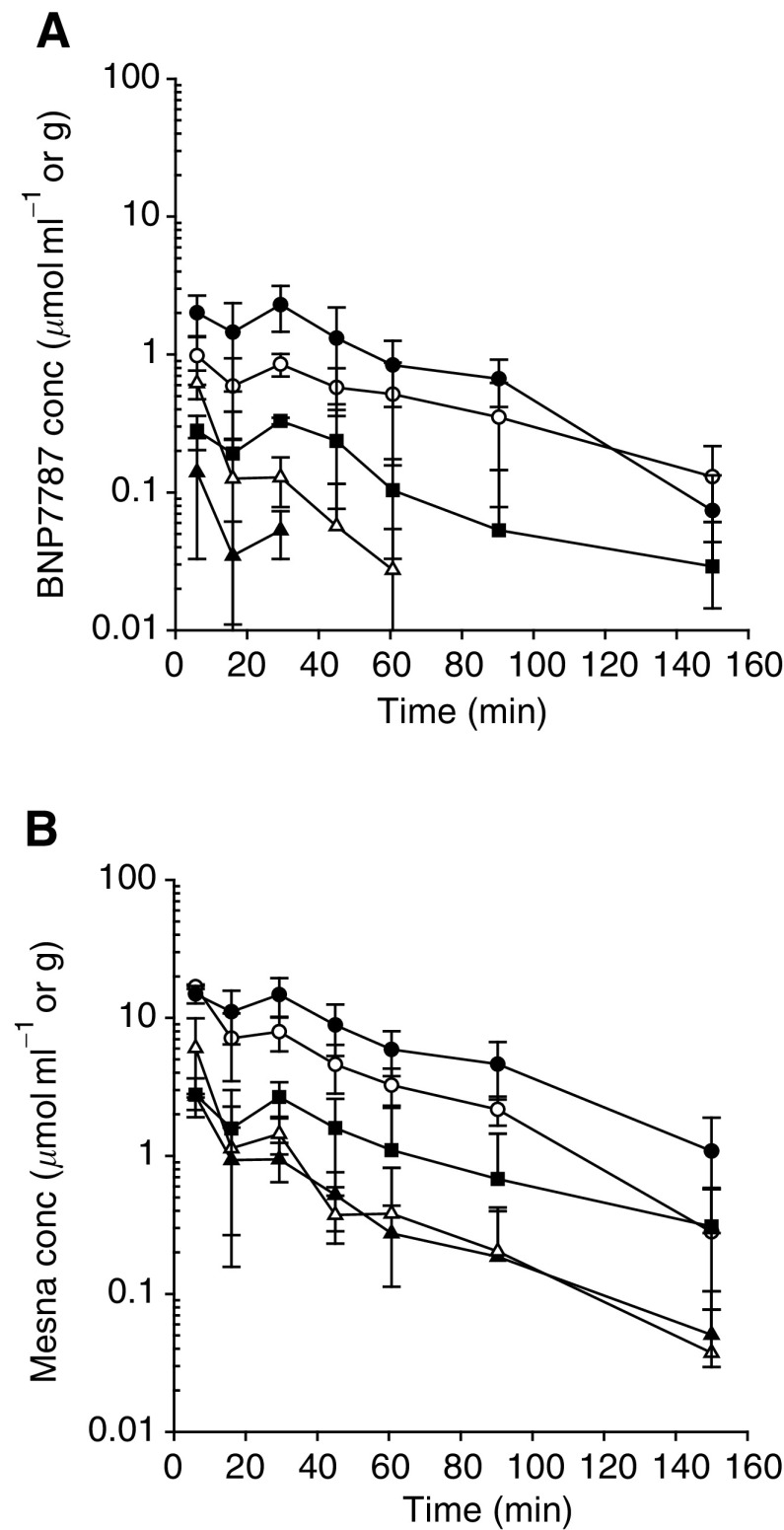
Semilogarithmic mean concentration (±s.d.)–time curves of BNP7787 (**A**) and mesna (**B**) in the kidney (•), plasma (○), tumour (▪), RBC (▵) and liver (▴), obtained after administration of 1000 mg kg^−1^ i.v. mesna in Fischer rats.

Following the i.v. administration of 1000 mg kg^−1^ mesna, the highest concentrations of mesna were observed at 5 and 30 min after administration in all sampled compartments (Figure 2a and b

). After 30 min, the concentrations of mesna gradually decreased. The highest concentrations of the metabolite BNP7787 were observed at 5 and 30 min following administration, indicating that the generation of BNP7787 from mesna was relatively rapid. Mesna and BNP7787 were detectable for at least 150 min in kidney, plasma and tumour, and mesna was also detectable in RBC and liver during this time period.

Comparison of the concentrations of the parent drug mesna with the metabolite BNP7787 revealed that the concentrations of mesna were much higher than the concentrations of BNP7787 in all sampled compartments. This was reflected by the AUCs of mesna, which were at least seven-fold higher than the AUCs of BNP7787 in plasma and all tissues sampled.

Comparison of the concentrations of mesna and BNP7787 between the sampled compartments showed that the concentrations of both compounds were the highest in kidney, followed by plasma>tumour>RBC>liver (Figure 2a and b). The kidney–plasma AUC ratios of mesna and BNP7787 were 1.7 and 2.1, respectively. In all other tissue compartments, the tissue–plasma AUC ratios of mesna as well as of BNP7787 were lower than 0.3. At 30 min after mesna administration, the mean concentrations (±s.d.) of mesna and BNP7787 in skeletal muscle were 665.1±209.1 and 30.3±17.6 nmol g^−1^, respectively. These concentrations were lower than any concentration of both compounds measured at that time point in plasma or the other sampled tissues.

### BNP7787 administration

**Figure 3:**
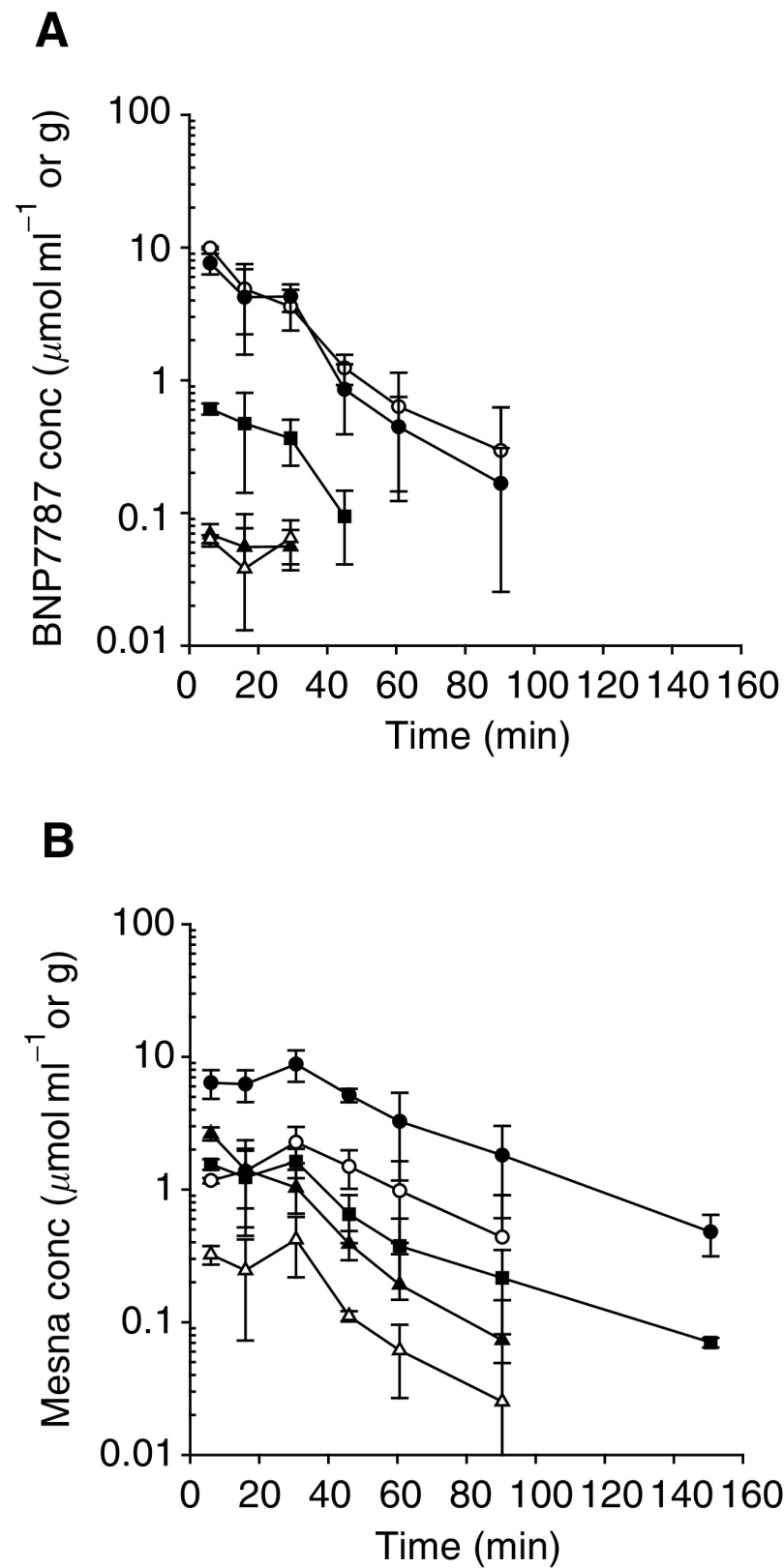
Semilogarithmic mean concentration (±s.d.)–time curves of BNP7787 (**A**) and mesna (**B**) in the kidney (•), plasma (○), tumour (▪), RBC (▵) and liver (▴), obtained after administration of 1000 mg kg^−1^ i.v. BNP7787 in Fischer rats.

Semilogarithmic mean concentration–time curves of BNP7787 and generated mesna after the i.v. administration of 1000 mg kg^−1^ BNP7787 are shown in Figure 3a and b

. The highest concentrations of BNP7787 were observed in all compartments at 5 min after administration. High peak levels of the metabolite mesna were maintained between 5 and 30 min after administration of BNP7787. Only in the liver the mesna concentrations decreased immediately after the maximal concentration was observed at 5 min after administration. BNP7787 could be detected for 90 min in the plasma and kidney, 45 min in tumour and only 30 min in RBC and liver. Mesna could be detected for at least 150 min in kidney and tumour, whereas in plasma, liver and RBC mesna could be detected up to 90 min after BNP7787 administration.

In plasma, the concentrations of the parent drug BNP7787 were higher than the concentrations of its metabolite mesna, as reflected by the AUC of BNP7787, which was almost two-fold higher than the AUC of mesna. In contrast, the AUCs of BNP7787 in the kidney, RBC, liver and tumour were, respectively, 2.7-, 11.4-, 39.8- and 5.1-fold lower than the AUC of mesna in these tissues.

The highest BNP7787 concentrations were obtained in plasma and kidney, whereas the concentrations of BNP7787 in liver, RBC and tumour were substantially lower (Figure 3a and b). This was also reflected by the tissue–plasma AUC ratios, which were 0.9 in kidney, 0.08 in tumour and 0.01 in RBC and liver. The highest concentrations of the metabolite mesna following BNP7787 administration were reached in the kidney and were much higher than the concentrations in plasma, as reflected by the kidney–plasma AUC ratio of 4.5. Mesna concentrations observed in the liver, RBC and tumour were lower than those in plasma (i.e. tissue–plasma AUC ratios of 0.5, 0.1 and 0.7, respectively). At 30 min after administration of BNP7787, the mean concentrations (±s.d.) of BNP7787 and mesna in skeletal muscle were 36.9±26.0 and 355.1±113.0 nmol g^−1^, respectively, which were lower than the concentrations observed in any of the sampled compartments at the same time.

### BNP7787 *vs* mesna administration

**Figure 4:**
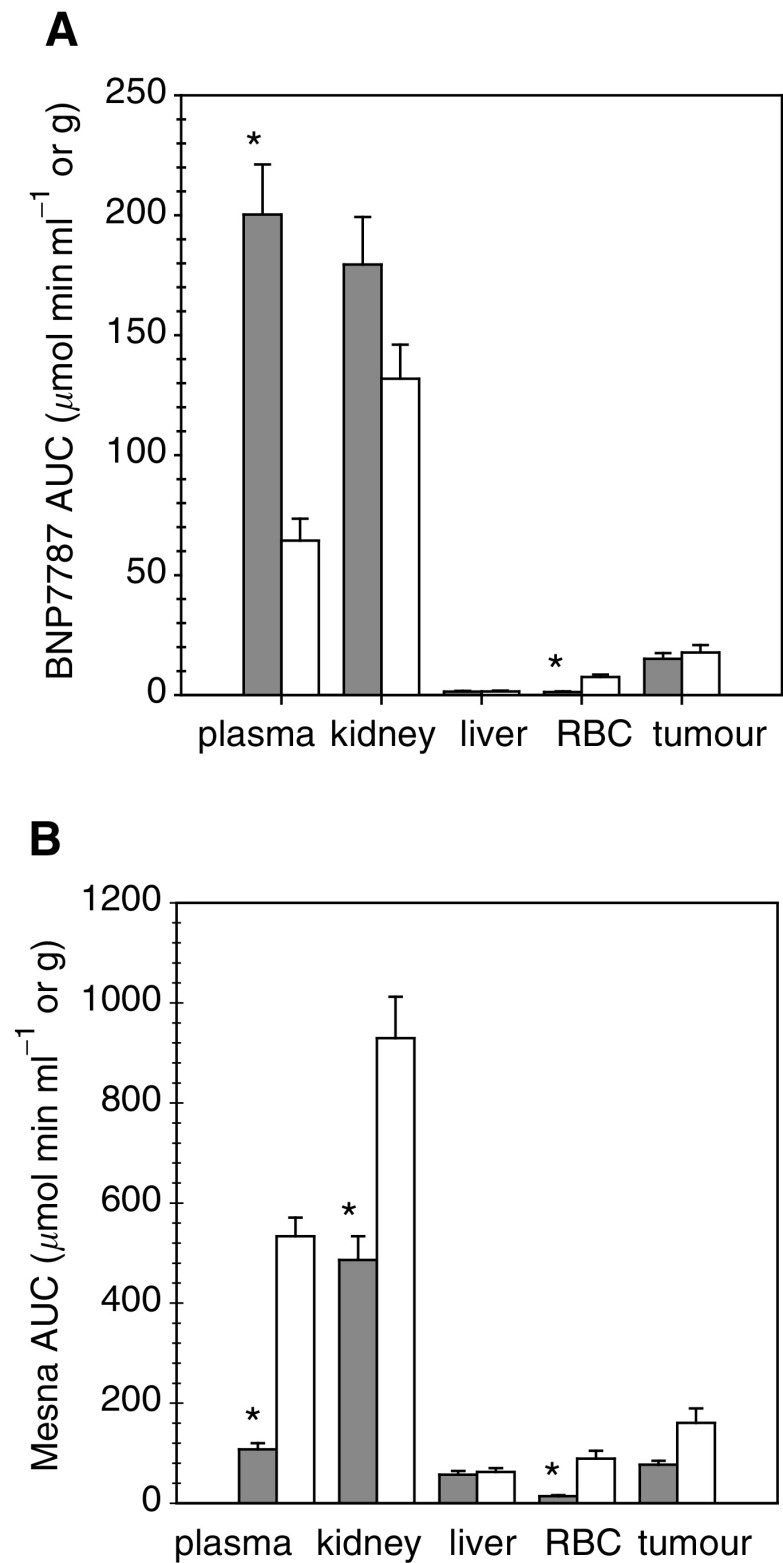
AUC values of BNP7787 (**A**) and mesna (**B**) in the plasma, kidney, liver, RBC and tumour from rats treated with 1000 mg kg^−1^ i.v. BNP7787 (grey bars) compared to the values obtained after 1000 mg kg^−1^ i.v. mesna (white bars). All AUC values were determined from the concentration–time curves shown in Figures 2 and 3 from 5 min after drug administration up to the time of the last measurable concentration or to the last sampling time, that is, 150 min. ^*^P<0.01.

For reasons of comparison, the AUC values of BNP7787 after BNP7787 and mesna administration are depicted in Figure 4a

, while those of (generated) mesna are shown in Figure 4b.

In plasma, the AUC of BNP7787 was more than three-fold higher after BNP7787 administration than after mesna administration (*P*<0.01, Figure 4a). A smaller, but not significant difference was observed in kidney. In contrast, a six-fold lower concentration of BNP7787 was measured in RBC after BNP7787 administration (*P*<0.01). The AUC values of BNP7787 in the other compartments (i.e. liver and tumour) were not significantly different following the administration of BNP7787 or mesna.

In plasma, the AUC of mesna was five-fold lower following BNP7787 administration than after mesna administration (*P*<0.01, Figure 4b). In kidney and RBC, a respectively 1.9-fold and 6.2-fold lower AUC of mesna was observed after BNP7787 than after mesna administration (*P*<0.01). Of interest, the AUC of mesna in the tumour was 2.1-fold lower following BNP7787 administration relative to mesna administration, although this was not statistically significant. In the liver, the absolute AUC values of mesna were similar after BNP7787 and mesna administration.

## DISCUSSION

Currently, BNP7787 is undergoing clinical development to study its potential as a chemoprotector against cisplatin-induced nephrotoxicity, as well as platinum- and taxane-induced neurotoxicity (Hausheer *et al*, 1998, 1999; Cavalletti *et al*, 1999; Boven *et al*, 2002). In the kidneys, BNP7787 is postulated to undergo selective conversion into mesna, which can locally detoxify cisplatin. Studies in tumour-bearing rats (Hausheer *et al*, 1998) have confirmed the selectivity of BNP7787 administration that resulted in complete protection against cisplatin-induced nephrotoxicity, as documented by histopathological examination, with full preservation of the antitumour activity of cisplatin. In contrast, reduction of the antitumour activity of cisplatin was observed in tumour-bearing rats and nude mice receiving mesna (Hausheer *et al*, 1998; Boven *et al*, 2002). The results in this study showed that high concentrations of BNP7787 and mesna were observed in the plasma and kidney of WARD tumour-bearing rats receiving either BNP7787 or mesna. In all other tissue-sampled compartments, the AUC values of BNP7787 and mesna were at least 5.5-fold lower than the corresponding values in kidney. After mesna administration, the plasma concentrations of mesna were substantially higher than those after BNP7787 administration and were comparable to the concentrations of mesna obtained in the kidney after BNP7787 administration.

For pharmacokinetic comparison of the various samples described herein, AUCs to infinity are preferred. However, due to the large variations in some *C̄*_*i*_ values, it was not possible to perform reliable AUC extrapolations to infinity for all tissues. Therefore, AUCs were calculated from 5 min after drug administration up to the last measurable concentration or to the last sampling time, that is, 150 min (AUC^5−*t*^). The quality of the latter approach is illustrated by calculating the AUC^5−∞^ values of the smooth concentration–time curves. In these cases, AUC^5−∞^ values were less than 13% higher than the corresponding AUC^5−*t*^ values. AUC^5−∞^ values of BNP7787 in the liver after mesna or BNP7787 administration and in RBC after BNP7787 administration differed much more from the calculated AUC^5−*t*^ values. This large variation was due to the limited number of time points, with concentrations of BNP7787 above the lower limit of quantification in these compartments. In addition, by using AUC^5−*t*^, it is possible to calculate an s.d. for AUC^5−*t*^, which is not possible for AUC^5−∞^. The aforementioned data show that the obtained AUC^5−t^ values are reliable values, which we can use for comparative pharmacokinetics.

Mesna has been shown to attenuate the antitumour activity of cisplatin, whereas administration of its disulphide BNP7787 did not (Hausheer *et al*, 1998; Boven *et al*, 2002). This distinction between the two compounds can possibly be explained by the higher plasma and tumour levels of mesna after administration of mesna, since mesna is more reactive with (hydrated) cisplatin than its disulphide BNP7787 (Leeuwenkamp *et al*, 1991; Verschraagen *et al*, 2003a). High concentrations of mesna were observed in plasma after administration of 1000 mg kg^−1^ mesna i.v. The AUC of mesna in plasma after mesna was substantially higher (five-fold) than the AUC of mesna after BNP7787 administration (*P*<0.01). In the tumour, the concentrations of mesna after mesna administration were also higher than after BNP7787 administration. The two-fold higher AUC value of mesna in tumour after mesna administration was, however, not statistically significant and was at least three-fold lower than the AUC value of mesna in plasma after mesna administration. The high concentrations of mesna in plasma after mesna were comparable with the high mesna concentrations obtained in kidney after i.v. administration of 1000 mg kg^−1^ BNP7787. This dose of BNP7787 has been shown to completely prevent cisplatin-induced nephrotoxicity in rats (Hausheer *et al*, 1998), suggesting that the observed high concentrations of mesna in kidney were capable of inactivating (hydrated) cisplatin before the occurrence of toxicity. Since mesna concentrations in plasma after mesna were comparable to the high inactivating mesna concentrations in kidney after BNP7787, this can likely explain why mesna administration can reduce the antitumour activity of cisplatin. Controlled pharmacokinetic studies of cisplatin in the presence or absence of BNP7787 or mesna are necessary to provide the conclusive evidence that mesna administration lowers the intact cisplatin concentration by forming cisplatin-mesna adducts in plasma, thereby reducing the antitumour activity, whereas BNP7787 administration does not.

BNP7787 administration has been shown to completely protect the kidneys against cisplatin-induced toxicity in rats (Hausheer *et al*, 1998). We measured high concentrations of BNP7787 and mesna following administration of BNP7787 in kidney. The AUC of mesna in kidney was approximately 4.5-fold higher than the value in plasma, whereas the AUC of BNP7787 was grossly similar for the two compartments. This finding is consistent with *in vitro* observations that in plasma the reduction of BNP7787 into mesna is very limited in contrast to the large reduction of BNP7787 observed in the cytosol fraction of the kidney cortex (Ormstad *et al*, 1983). The high kidney concentrations of BNP7787 and mesna are in contrast to the low concentrations of both compounds observed in skeletal muscle, liver, RBC and WARD tumour. These results clearly indicate that the hydrophilic BNP7787 and its metabolite mesna undergo negligible uptake by these tissues. The high concentrations of BNP7787 and mesna in the kidney confirm our hypothesis that the selectivity of BNP7787 in protecting the kidney against cisplatin-induced toxicity (Hausheer *et al*, 1998; Boven *et al*, 2002) is based on the preferential uptake of BNP7787 and its local conversion into the thiol mesna in the kidney (Ormstad and Uehara, 1982; Brock *et al*, 1984; Ormstad and Ohno, 1984). The high local concentrations of mesna in the kidney can inactivate (hydrated) cisplatin before the occurrence of toxicity by forming nontoxic platinum complexes. In addition, mesna can protect the kidney by acting as a scavenging agent for reactive oxygen species (Bast *et al*, 1987; Mashiach *et al*, 2001), thereby preventing lipid peroxidation which might be involved in cisplatin-induced nephrotoxicity (Sadzuka *et al*, 1992). Further research should help to further elucidate the precise mechanism of nephroprotection by BNP7787.

Studies in rats showed that the dose of cisplatin could be safely increased from 6 to 9 mg kg^−1^ when preceded by 750 or 1500 mg kg^−1^ BNP7787 i.v. (Hausheer *et al*, 1998). In nude mice, however, weight loss increased when the dose of cisplatin increased from 5 to 8 mg kg^−1^ in the presence of 1000 mg kg^−1^ BNP7787 (Boven *et al*, 2002). The pharmacokinetics of BNP7787 and mesna in the plasma of rats were compared with that in nude mice (Boven *et al*, 2002) to find a possible explanation for the difference in protection by BNP7787 between the two species. The relative amounts of the metabolite mesna present in the circulation after BNP7787 administration seems to vary between the two species. In nude mice, the AUC of mesna in plasma was approximately 24% of the AUC of BNP7787 (Boven *et al*, 2002), whereas in rats the AUC of mesna was approximately 50% of the AUC of BNP7787. This suggests that, in contrast to rats, small amounts of mesna are generated in nude mice. Furthermore, in rats, the AUC^5−60^ in plasma was approximately 5.2-fold higher for mesna and 2.7-fold higher for BNP7787 than the AUC^5−60^ values measured in nude mice after an equivalent dose of 1000 mg kg^−1^ BNP7787. These results indicate that, in comparison to mice, the administration of BNP7787 in rats results in higher concentrations of BNP7787 and mesna in plasma, but most probably also in kidney. This observation may partially explain the greater protection by BNP7787 against cisplatin-induced toxicity in rats than mice.

In conclusion, after BNP7787 administration high concentrations of its metabolite mesna, which is more reactive with (hydrated) cisplatin than its disulphide, were observed only in the kidney. In contrast, after mesna administration, the reactive thiol mesna was found in high concentrations in the kidney as well as in plasma. The substantially higher concentrations of mesna in plasma following mesna administration may explain why mesna administration results in reduced antitumour activity of cisplatin and why BNP7787 administration exerts no effect on antitumor activity as reported previously (Hausheer *et al*, 1998; Boven *et al*, 2002). The pharmacokinetic results confirm the selective protection of the kidney by BNP7787, which makes BNP7787 a promising chemoprotectant. In that respect, we have completed a phase I study on BNP7787 preceding cisplatin in cancer patients, demonstrating that BNP7787 was relatively nontoxic up to high doses of 41 g m^−2^ (Verschraagen *et al*, in press). Furthermore, at the higher dose levels of BNP7787 (18.4–41 g m^−2^), there appeared to be no mutual pharmacokinetic interaction between BNP7787 and intact cisplatin, which was consistent with the observation that several patients experienced objective tumor responses with cisplatin preceded by BNP7787 administration (Verschraagen *et al*, in press). BNP7787 is currently under investigation in three randomised studies to determine its safety and efficacy as a protective agent in combination with paclitaxel alone and in combination with cisplatin plus paclitaxel.
